# Risk of bias assessment tool for systematic review and meta-analysis of the gut microbiome

**DOI:** 10.1017/gmb.2023.12

**Published:** 2023-08-18

**Authors:** Thomas Lampeter, Charles Love, Trien T. Tang, Aditi S. Marella, Hayden Y. Lee, Armani Oganyan, Devin Moffat, Anisha Kareem, Matthew Rusling, Aubrey Massmann, Melanie Orr, Christian Bongiorno, Li-Lian Yuan

**Affiliations:** 1 New York Institute of Technology College of Osteopathic Medicine, Glen Head, NY, USA; 2 Des Moines University College of Osteopathic Medicine, Des Moines, IA, USA

**Keywords:** bias, microbiome, metaanalysis, systematic review

## Abstract

Risk of bias assessment is a critical step of any meta-analysis or systematic review. Given the low sample count of many microbiome studies, especially observational or cohort studies involving human subjects, many microbiome studies have low power. This increases the importance of performing meta-analysis and systematic review for microbiome research in order to enhance the relevance and applicability of microbiome results. This work proposes a method based on the ROBINS-I tool to systematically consider sources of bias in microbiome research seeking to perform meta-analysis or systematic review for microbiome studies.

## Introduction

The most common experimental design used to evaluate the effects of gut microbiome (GMB) genomic or taxonomic post-exposure remodelling has been cohort studies using either animal or human models. Randomised controlled trials (RCTs) for microbiome interventions are less common because we are still characterising microbiome post-exposure remodelling to identify promising markers or targets for microbiome intervention that would warrant subsequent evaluation by RCTs. Therefore, results from a systematic review with quantitative or pooled meta-analysis are essential in identifying candidates for RCTs.

A diligent risk of bias (ROB) assessment is a key step in systematic review or meta-analysis to determine the likelihood that features of the study design or conduct of the study will give misleading results. GMB research is highly heterogeneous in its methods, reporting, and attempts to address bias. This manuscript and its associated rubric (Table 1) are based on the Risk of Bias in Non-randomised Studies – of Interventions (ROBINS-I) tool, and are meant to be used as a GMB-specific adjunct to ROBINS-I. This manuscript and its associated rubric together form a tool that was developed to help standardise ROB assessment in metanalyses and systematic reviews of GMB studies. A small-scale validation test by first-time ROB assessors produced consistently similar ROB determinations, suggesting that this tool can successfully guide consistent ROB determinations. This tool may allow for improved ROB assessment when evaluating studies for metanalyses and systematic reviews of the GMB.

**Table 1.** The rubric of domains and subdomains of bias with signalling statements to guide risk of bias assessment of gut microbiome studies.

Domain	High ROB	Moderate ROB	Low ROB
Confounding			
1. Demographic differences	– Age: consistently different between study arms – Sex: consistently different between study arms	– Age: mixed ages within a study arm, but equal in distribution between study arms – Sex: mixed sexes within a study arm, but equal in distribution between study arms	– Age: consistently similar between study arms – Sex: consistently similar between study arms
2. Habitat stability	– No acclimation period, or acclimation period <2 days – Acclimation period included in the interventional period	– Acclimation period ≥2 days but <5 days	– Acclimation period ≥5 days and < 9 weeks
3. Genotype, familial, and source differences	– Significantly different subject genotypes between study arms (where genotype effect is not the target of investigation) – Non-matured animal models from different litters and/or mothers without random assortment into study arms – Comparison of animal subjects from different source or vendor between study arms	– Animal subjects from same vendor, but from separate and temporally spaced orders without random assortment into study arms	– Adequately similar genotypes used between study arms (where host genotype effect is not the target of study) – Animal subjects from same litter – Animal subjects from same vendor and same order – Adult animal subjects from different litters/mothers/vendors randomly assorted into study arms
4. Extreme diet	– No statement of dietary standards or documentation of dietary variation – Major deviations from stated diet	– Study uses human subjects outside of a highly controlled environment (for example an inpatient healthcare setting)	– Use of identical diet between study arms where diet is not the target of study
5. GMB normalisation	– No documented means of verified GMB normalisation methods employed prior to intervention – Use of different normalisation methods between study arms or use of non-validated technique	– Antibiotic normalisation employed <7 days prior to intervention	– Antibiotic normalisation employed ≥7 days prior to intervention – Validated technique of GMB normalisation employed
Selection Bias			
1. Extreme genotype	– Subjects of known extremely different genotypes – Subjects with no established history of use	– Syngeneic subjects with limited established history of use	– Syngeneic subjects with established history of use
2. Randomisation or demographic balancing sufficiently applied	– Absence of both RCT and implementation of consistent host demographic across study	– Utilisation of RCT or implementation of consistent host demographics across study	– Utilisation of RCT and implementation of consistent host demographics across study
Classification of Intervention			
1. Intervention bias	– Differential misclassification of intervention or test subject based on exposures present or suspected	– n/a	– Differential misclassification of intervention or test subject based on exposures absent or not suspected
2. Validation of method	– No validation that treatment method produces intended effect – Use of new method without internal validation	– n/a	– Documented use of validated methods – Use of a new method with adequate internal validation
Deviation from Intervention			
1. Deviation from intervention	– Large deviations to protocol without adequate time stamps, rationale, and limitations noted	– Slight deviations to protocol with adequate time stamps, rationale, and limitations noted	– Intervention successfully carried out without protocol deviation
Missing Data			
1. Cause or category of missing data	– Does not address missing data qualitatively or quantitatively – Or, does not ensure to readers the absence of missing data	– Acknowledges missing data qualitatively or quantitatively – Inadequate MAR/MNAR distinction or proper statistical correction	– Addresses missing data qualitatively or quantitatively, or ensures the absence of missing data – Adequate MAR/MNAR distinction or proper statistical correction
2. Subject dropout	– Subject drop-out exceeds 20%	n/a	– Subject drop-out is equal to or less than 20%
3. Sequencing depth and sampling zeroes	– Does not address sampling zeroes with statistical correction	n/a	– Addresses sampling zeroes with statistical correction
Measurement of Outcomes			
1. Sample collection	– Inconsistent collection time	– Animal models: Collected at same time, not in the morning – Human models: Inconsistent collection time, but preserved immediately after defecation	– Animal models: Collected at same time, in the morning – Human models: Consistent collection time and preserved immediately after defecation
2. Blinding	– No double blinding when the primary measurement is subjective	n/a	– Primary outcome is objective measure such as genetic sequencing not subject to bias by the subject or investigator – Primary outcome is subjective and double or greater blinding employed
Reporting of Results			
1. Selection of reported results	Any of: – Omission of stated outcomes that are unfavourable or statistically insignificant – Addition of outcomes not in the initial protocol – Results reported are only on a subset of data – Changing outcome(s) of interest	– Any of the above, but with valid and satisfactory explanation provided	– Inclusion of relevant null and significant findings as stated in the protocol

## Using this tool

This manuscript and its associated rubric provide a framework for assessing ROB specific to GMB research. This tool strives to provide insight and reduce variability between individual researchers and groups conducting systematic reviews of the GMB. We do not seek to suggest best practices. Instead, we aim to indicate potential sources of bias that may significantly impact GMB studies and are thus vital when considering the strength of evidence for systematic review and meta-analysis. The essential criteria in this manuscript are summarised in Table 1, which was compiled to act as a rubric in guiding ROB determination.

Table 1, “the rubric,” guides the determination of low, moderate, or high ROB across seven domains. In each cell of the rubric, there are signalling statements to help guide low, moderate, or high ROB determination in that domain. Two additional ROB determinations are not included on the rubric as they are to be used at the judgement of the person assessing ROB in a study. They are “critical ROB” and “no information.” Critical ROB can be determined when a reviewer believes a study to be too problematic to provide useful evidence on the effect of an intervention. As such, a study determined to be of critical ROB in any one domain should not be included in any synthesis. A determination of no information applies to domains where there is no clear evidence of a critical ROB *and* a lack of information to judge ROB otherwise.

## Confounding

### Demographic differences

Important demographic considerations in GMB studies are sex and age. Substantial differences in the gut microbiota are attributable to sex differences in mammals (Org et al., 2016; Kim et al., 2020). Because of this, any study which includes one sex in one arm and a different sex in another should be classified as having a high ROB. In addition to the ROB from sex, other demographic factors may also introduce confounding bias into the studies being examined. The GMB changes with age across numerous conditions, disease models, and species impacting microbial diversity and biome composition (Ticinesi et al., 2019; Liu et al., 2020). Therefore, age differences between cohorts and study arms should be assessed. If the study being examined uses organisms of one age in one arm and a different age in a second arm, it should be classified as having a high ROB. The age gap which introduces significant confounding bias, varies by organism. An example of an age gap that would introduce a high ROB is 8-week-old mice versus 1-year-old mice (Yoon et al., 2021).

### Habitat stability

The habitat in which organisms are kept substantially impacts their GMB (Singh et al., 2021). Mice, common subjects of microbiome research, are known to have highly variable microbiomes on arrival at a facility, likely because of transportation stress on the microbiome itself and the immune system and hormonal functions of the host organism (Capdevila et al., 2007; Montonye et al., 2018; Lipinski et al., 2021). Studies that do not allow for microbiome stabilisation before research begins risk confounding bias due to a lack of habitat stability. Organisms should be acclimated to the study condition before baseline measurements or interventions are performed. However, an extensive acclimation period risks microbiome drift occurring due to the increasing age of the organism or other unknown factors, so habitat stabilisation must be time-limited (Hoy et al., 2015). Additional bias would also be introduced if the acclimation period is included in the interventional period of the research.

### Genotype, familial, and source differences

Subject genotype, degree of familial relation, and in the case of animal models, the source can significantly impact GMB composition. Differences in the genotype of animal models have been found to impact the diversity and abundance of organisms (Campbell et al., 2012; McKnite et al., 2012; Leamy et al., 2014). For this reason, if the study being evaluated uses organisms of significantly different genotypes, such as the use of different strains of mice from the Collaborative Cross, where the effect of genotype difference is not the target of the study, it should be classified as having a high ROB. Suppose the study uses a similar genotype between treatment groups, such as the same strain of inbred animal model or monozygotic twin subjects. In that case, it should be considered a low ROB for confounding due to the genotype effect.

Regarding familial relation, genetically related subjects have been demonstrated to share a core of similar GMB for up to three generations in the female line (Turnbaugh et al., 2008; Valles-Colomer et al., 2021). With animal models, breeding within familial relations is often used to maintain genotypically and GMB homogeneity (Hufeldt et al., 2010). A caution regarding inbreeding is that while selective breeding between siblings can create a more stable and uniform GMB composition, the effects of genetic drift can also introduce confounders across multiple generations that may affect experimental reproducibility with subsequent generations (Laukens et al., 2016).

Additionally, with animal models, an organism’s litter of origin impacts the gut microbiota (Fujiwara et al., 2008; Vilson et al., 2018). This may relate not only to parent genetics but also to the host of maternal factors that can affect the development of progeny GMB, including mode of delivery, maternal diet, maternal stress, and maternal antibiotic use (Bailey et al., 2004; Friswell et al., 2010; Stokholm et al., 2014; Golubeva et al., 2015; Walker et al., 2017; Zhang et al., 2021). For these reasons, if the study being examined utilises organisms from differing litters (from separate mothers or separate deliveries from the same mother) that have not yet reached their mature adult development and are not randomly assorted between research arms, it should be classified as having a high ROB. Suppose a study uses organisms from the same mother and litter or randomly assorts progeny from different mothers and litters. In that case, it should be classified as having a low ROB.

Regarding sourcing of animal models, subjects sourced from different vendors have substantial differences in GMB at baseline (Rasmussen et al., 2019; Wolff et al., 2020; Long et al., 2021). The microbiological or physiological basis of these effects is unknown but may be due to differential exposures to environmental or infectious factors between vendors (Mandal et al., 2020).

### Extreme diet

Dietary differences have been shown to alter the abundance of most gut microbes (Daniel et al., 2014; Do et al., 2018; Ang et al., 2020; Li et al., 2021). Because of this, maintaining the diet of interest is essential to avoid introducing confounding bias to the study. However, it may not always be possible to strictly control diet. This is especially relevant to clinical studies involving humans. In this situation, an evaluation of bias must note how a study documented these diet variations.

### GMB normalisation

It is important to assure organisms being studied in research have similar baseline GMB. This allows for more definitive inference as to the effect of the intervention. Several strategies have been used to make the GMB as similar as possible over time. Removal of the entire GMB through the use of germ-free mice can allow for the artificial seeding of a select group of organisms (Yi and Li, 2012; Kennedy et al., 2018). However, the use of these mice necessarily limits the generalizability of a study. For this reason, research often uses organisms with populated GMBs and relies instead on antibiotics to homogenise the microbiome. The use of antibiotics introduces additional risks of bias which must be considered when evaluating a study (Theriot et al., 2016). The most significant ROB arises from beginning the intervention of interest before the gut microbiota has stabilised after normalisation with antibiotics. The GMB continues to fluctuate unpredictably for long periods following antibiotic administration (Merenstein et al., 2021). This variance has been found for at least a year after antibiotic usage in humans and for times ranging between 1 week and 16 weeks in mice depending on the length of the course of antibiotics used (Rashid et al., 2015; Elvers et al., 2020; Zhu et al., 2021). However, short, or single doses of antibiotics such as those often used to normalise the microbiome allow for substantial stabilisation of the GMB within 7 days (Gu et al., 2020).

A third method used to standardise the GMB is to intermix the bedding of multiple cages and then redistribute it (Miyoshi et al., 2018). This method is less invasive than antibiotic usage and has a lower risk of long-term impact on the GMB than the use of antibiotics. The use of homogenisation of the bedding allows for similar microbiomes to develop in more mice than can be practically housed in a single cage, where the organisms also share all of their bedding (McCafferty et al., 2013).

Because of the impact of different methods of GMB normalisation, it is critical to note the method that was used to normalise the GMB and how long before the intervention this normalisation was completed.

## Selection bias

### Extreme genotype

Host genotype shows a stable and heritable impact on GMB composition (Goodrich et al., 2016). In the context of GMB research, extreme genotype selection refers to the selection of GMB subjects with genotypes that vary significantly between subjects within a study. Selection of subjects with identical or similar genetic makeup limits genotype confounding effects. A subject with an established history of use along with maximised genetic correlation can be considered a low risk of selection bias. For example, while inbred Balb/C mice do have an extreme genotype, they also have a long-established history of use in immune modulation studies with their known Th2 immune response wherein they exhibit low IFNy and high IL-4 production (Mills et al., 2000; Watanabe et al., 2004; Khan et al., 2022). Furthermore, prior literature has established the correlation between subject genetics and variation in the GMB population and subsequent disease states (Xu et al., 2020).

### Randomisation or demographic balancing sufficiently applied

Randomisation is essential in ensuring subject-level differences between participants in the intervention and control groups can be attributed to chance alone. It is a standard method that attempts to create the necessary pre-intervention equivalence between groups, allowing for conclusions based on the effect of the intervention. In trials where randomisation was not appropriately utilised, the outcome was overestimated by up to 40% compared to trials where randomisation was utilised (Suresh, 2011). If randomisation was not applied, implementing demographic balancing is an appropriate measure to ensure adequate control and intervention arms distribution. Any demographic balancing performed should be sufficiently described in the study. This method focuses on ensuring each group is demographically balanced at baseline to lessen the difference between groups and utilise randomisation if no subject background information is available (Saint, 2015). Both randomisation and demographic balancing can be applied to human and animal model studies. For example, in studies utilising syngeneic mice, randomisation must be performed outside the scope of human intervention in that random number generators should assign mice numbers which can then correlate to intervention and control groups, hence this places randomisation outside the scope of human influence, limiting bias to a maximum degree. In syngeneic animals, demographic balancing would have a limited impact on the bias, however, wherein studies utilise genetically unrelated animals, the need for implementation of both randomisation and demographic balancing is necessary for limiting substantial bias (Hirst et al., 2014). Similar principles apply in human studies. Given a majority of human studies utilise genetically unrelated subjects, randomisation is required to avoid the high ROB. In human studies, a step beyond randomisation should be taken, that is, implementing blinded randomisation with a description of the randomisation protocol to give the reader the ability to discern breaks in randomisation or similar bias control methods within the study (Chalmers et al., 1983).

## Classification of intervention

### Intervention bias

Bias in intervention can occur when interventions or outcomes are inappropriately selected for or measured. In non-differential misclassification, test subjects’ exposures are misidentified, and they are categorised into the wrong group (McCoy, 2017). This misclassification can dilute the effect of the intervention causing effect estimates to favour the null (LaMorfe, 2016). The probability of non-differential misclassification is equal across all groups. Bias may be reduced by ensuring a proper background check on test subjects and equalising any differences. On the other hand, differential misclassification occurs when the misclassification of exposure or outcome is not equal between subjects and is less easily predictable in whether it will bias results towards or away from the null. Therefore, the probability of assigning subjects to the wrong group differs based on the individual. This may also introduce recall bias towards recalling specific exposures because the subject has the disease state versus a subject that does not. In GMB studies, this may present in the form of researchers explaining results that show a significant effect as attributed to specific causes but leaving out explanations for non-significant results. Because this type of misclassification is more applicable in case studies, it is less relevant for animal studies but can be prominent in human studies (Spencer et al., 2018).

### Validation of method

The establishment of an effective intervention is imperative for a successful study. Before the experiment, researchers must verify that their chosen intervention method will produce the intended effect. In studies where this is not done, the produced results may or may not be relied on because the protocol was never validated. Verification can be internal (tested and proved by the researchers) or external (via other established studies). If the study calls for a particular disease state to be expressed, it must be validated that the test subjects have the disease state. In studies that call for a specific procedure, there can be potential bias in how the readers know the procedure was correctly obtained if it is not reported. For example, in microbiome hypertension studies, animal subjects were tested based on blood pressure measurements by a well-established method, tail-cuff plethysmography (Marques et al., 2019). If a lesser-known and validated method was used, it could introduce a high ROB if researchers did not verify that their method was accurate. When testing for the effect of a disease state as influenced by the microbiome, it is helpful to transplant the experimental group microbiome into a germ-free animal model to confirm the effect. This reduces an intermediate ROB by demonstrating that the effect of the intervention is associated with the levels of change in the microbiome (Gottfredson et al., 2015).

## Deviation from intervention

It is well understood that experiments that deviate from their initial protocol have an increased potential for bias in their study should they decide to include data prior to the deviation. Therefore, all deviations from the protocol should be well documented with time stamps, and the data included in the study should also include the time at which it was collected – either post-protocol or pre-protocol addendum. Rationale and limitations should also be included should researchers decide to include data from any time the protocol was different.

## Missing data

Missing data is prevalent in many academic disciplines, from the social to biomedical sciences, and may contribute to bias in any given study. GMB research likewise suffers from inadequate consideration of missing data and the statistical methods to address it. To begin, two types of missing data should be distinguished: missing data due to patient drop-out in clinical, longitudinal studies and missing data as a result of inadequate sequencing depth leading to “false zeroes” in the microbiome genetic data. Both have the potential to increase ROB.

### Cause/category of missing data

Missing data falls into multiple categories based on the mechanism of missingness: Missing Completely at Random (MCAR), Missing at Random (MAR), and Missing Not at Random (MNAR) (Groenwold and Dekkers, 2020). These categories apply assumptions to missing data based on the cause. MCAR assumes that data is missing due to a factor entirely unrelated to the study. MAR assumes data is missing due to observed variables relevant to the study. MNAR assumes data is missing based on unknown or not quantifiable variables to the authors. MAR and MNAR are most relevant to clinical research, specifically in regard to patient drop-out, including clinical GMB trials (Pugh et al., 2021). Sampling zeroes in microbiome data are a more generalised form of missing data but are primarily reminiscent of MAR (Kaul et al., 2017a, 2017b). Each of these areas will be further discussed in the following sections. Under MAR, studies may utilise various statistical imputation techniques to replace missing data, though the most well-known and effective method is multiple imputations (Spineli et al., 2015). With MNAR, various statistical modelling techniques may address missing data. Such techniques are further discussed in relation to GMB studies in section “Sequencing depth and sampling zeroes.” The distinction between MAR and MNAR also indicates whether bias related to missing data is entirely removable in analysis – the former can, while the latter cannot (Mack et al., 2018). This should not be confused with the notion that MNAR assumptions immediately denote a study as biased. If the missingness in MNAR or MAR is independent of the outcome, then the study may be unbiased in regard to missing data. Thus, a study with MNAR data is not necessarily high ROB.

Notably, a significant number of studies do not clearly state the mechanism of missingness or adjust for missing data (Carpenter and Smuk, 2021). It is important that studies distinguish the mechanism of missingness or explain relevant missing data. If a study does not acknowledge missingness in data or ensures the absence of missing data, the study may be considered high ROB. If a study acknowledges missing data but does not adequately address it through MAR/MNAR distinction and proper statistical techniques related to its missing data category, then the study may be considered intermediate ROB. If a study demonstrates all of this, it may be considered low ROB.

### Subject drop-out

Missing data in the form of patient drop-out has a marked effect on statistical power, type 1 error, and various outcome measures (Thompson et al., 2011; Fiero et al., 2016; Cai et al., 2020). In traditional clinical research, missing data has a clear effect on useful measures, such as relative risk and risk ratio calculations. Further, although researchers attempt to minimise drop-out and its statistical effects, drop-out ratios were reported to be greater than 40% depending on the study and the degree of unpleasantness in medical interventions to the patient (Schnicker et al., 2013; Li et al., 2021). Consequently, it has been proposed that a 20% drop-out ratio is reasonable (Furlan et al., 2009; Cramer et al., 2016). Interestingly, it has been shown that faecal sampling of patients in GMB studies has not been a significant reason for drop-out, suggesting typical sources of patient non-retention (Vandeputte et al., 2017). The effect of drop-out on statistical measures is expected to be the same in clinical GMB trials. Despite drop-out being common in clinical studies, its effect on outcome measures involving microbial compositional data (e.g., beta diversity) is not currently well described in clinical GMB studies. However, it is expected that such measurements relying on consistent analysis from a wide array of samples will be biased if there is an inadequate sampling size.

The effect of bias comes into effect when there is an interpretation between samples, in that missing data prevents consistent interpretation of genetic data through a larger body of samples. For example, microbiome samples stratified by disease state versus control should be held to higher statistical power, similar to traditional clinical studies. Yet, the complexity of GMB genetic analysis often prevents large sample sizes from being a practical implementation due to costs unless utilising less-expensive protocols such as those involving qPCR to monitor microbial composition at high taxonomic levels (i.e., phyla) (Koliada et al., 2020). Some studies demonstrate shallow shotgun metagenomic sequencing as an alternative methodology for large, longitudinal GMB studies (Xu et al., 2021). Nonetheless, making interpretations in GMB data between samples stratified by host conditions may need to be more consistent and accurate when samples are unavailable from a patient drop-out. Based on the literature of other areas in clinical research as discussed, it is again reasonable to assert that drop-out will influence outcome measures if authors make interpretations across hosts of varying condition states.

Due to few clinical studies analysing the effect of drop-out on GMB outcomes, it is reasonable to use a 20% patient drop-out ratio, as many clinical trials traditionally utilise. GMB studies that have a high patient dropout are considered high ROB. GMB studies that have low patient drop-out are considered low ROB.

### Sequencing depth and sampling zeroes

GMB researchers should consider sequencing depth as a contributor to missing data and subsequent bias. It is established that low-sequencing depth (2000 single-end reads per sample) can adequately predict the same diversity patterns as high-depth sequencing (on the scale of millions of reads per sample) (Caporaso et al., 2011; Lundin et al., 2012; Xiao et al., 2018). Experiments that quantify GMB outcome measures (like alpha and beta diversity) should utilise the same depth for all samples. Bias would be introduced if different sequencing depths are used for a set of samples. It should be noted, however, that false zeroes influence microbiome genetic data at both high and low depth. While true zeroes (or biological zeroes) represent true taxonomic absences, false zeroes (or sampling zeroes) represent a lack of sequencing depth to adequately detect certain microbial taxa. Notably, low sequencing depth, as is often the case of 16S rRNA sequencing, may not detect low abundance taxa or low taxa (subspecies) due to lower resolution. Though whole genome sequencing (WGS), such as shotgun metagenomic sequencing, utilises high sequencing depth to sequence entire genomes, sampling zeroes still persist (Pereira-Marques et al., 2019).

At the time of writing, this issue of zero-inflation – or the excess of sampling zeroes at high and low depth – and the resulting bias in GMB genetic data is an active area of research. Interestingly, relatively few studies utilise any statistical modelling to correct for such missing data. Yet, various modelling techniques were recently developed to address zero-inflation (Ha et al., 2020; Zhang et al., 2020; Deek and Li, 2021). Similar to modelling techniques, imputation is a method traditionally used to address missing data in the form of patient drop out, but a promising imputation method is recently available to also deal with GMB sampling zeroes. Previous studies showed an increase in Pearson correlation from 0.59 (between 16S and WGS in non-corrected data) to 0.64 (between 16S and WGS in corrected data) (Jiang et al., 2021 *).* There were also marked differences in mean and standard deviation of abundances per taxon between corrected and non-corrected data. This suggests greater homogeneity of samples across sequencing methods if imputation is utilised to correct data. However, as our article focuses on the role of bias in GMB research, we do not yet place best-practice recommendations for a particular method of missing data correction.

As of date, few GMB studies utilise statistical techniques to correct for sampling zeroes. Furthermore, common bioinformatics pipelines (such as QIIME2) do not incorporate such techniques into data-correction programs.

As such, the available literature suggests future GMB studies that do not consider sampling zeroes and lack a statistical technique for missing data correction may be considered high ROB. Studies that utilise missing data correction may be considered low ROB. These data correction methods, once more, include various modelling techniques or imputation.

## Measurement of outcomes

### Sample collection

Currently, there is no standard method for sample collection for GMB studies. While biopsy of the lower intestine provides a controlled sampling site and an accurate microbiota account, it is expensive, time-consuming, and unsuitable for healthy control groups. In contrast, the faecal collection is non-invasive and cost-effective (Tang et al., 2020). Thus, it is a standard sampling method in both clinical and research applications. However, faecal sample collection introduces temporal inconsistency which can be a source of bias.

Faecal samples collected at different times of the day are at risk for inaccurate representation of the absolute abundance of gut microbiota (Caporaso et al., 2011). Specifically for mouse studies, the snapshots of the microbiota provided by the faecal samples are more accurate and consistent within treatment groups when collected in the morning due to the nocturnal feeding nature of mice (Jones et al., 2021). For studies involving subjects with unpredictable and inconsistent bowel movements, samples should be preserved immediately after defecation as oxidation of the outer layer can alter the microbiota (Pepper and Rosenfeld, 2012). Specifically, *Firmicutes* and *Bifidobacteria* spp. are two known phylum that are unstable in the outer microenvironment when exposed to oxygen (Gorzelak et al., 2015). Therefore, to minimise the differential errors, the methods of measurement must be consistent between control and intervention groups.

### Blinding

In a GMB study, the primary outcome is based on definitive and objective genetic sequencing. Therefore, assessor bias is typically negligible, and a low ROB is expected (Higgins et al., 2022).

## Reporting of results

### Selection of reported results

Selective reporting of results can lead to biased interpretations of significance and or non-significance via particular selection of results from multiple outcome measures in estimating outcome effect. Bias in the selection of reported results can be difficult to detect without access to a protocol from which one can compare pre-specified intended outcomes of interest to the outcomes analysed in the published paper (Heneghan et al., 2019). Often, results are selected for significance, omitted for non-significance, or omitted for adverse effect of intervention (Dwan et al., 2013; Hedin et al., 2016; Van der Steen et al., 2019).

## Validation test

Four medical students with no prior experience in ROB assessment were recruited to test this tool by using it to independently assess ROB on three selected studies of similar length in a predetermined sequence (Wu et al., 2017; Mohammed et al., 2020; Saunders et al., 2020). Subjects were provided with the manuscript and ROB rubric. They were asked to track time to completion per study and complete the ROB rubric for each study. Subjects assessed ROB in an average of 44.75 minutes per study with time to completion generally decreasing from the first study assessed to the last study assessed.

**Figure 1.**
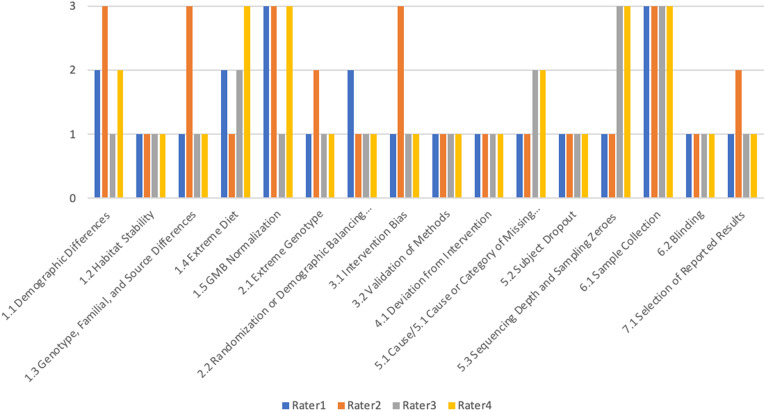
Inter-rater variability in ROB determinations by subdomain for validation test study 1 by Wu et al. (2017), where “1” on the *y*-axis indicates that the rater determined the study to be at low ROB for the subdomain indicated on the *x*-axis; “2” indicates medium ROB and “3” indicates a high ROB determination by the individual rater.

**Figure 2.**
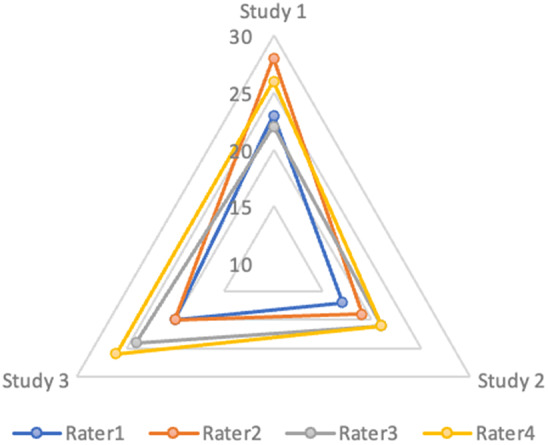
Inter-rater variability in ROB determinations by subdomain for validation test on study 2 by Mohammed et al. (2020), where “1” on the *y*-axis indicates that the rater determined the study to be at low ROB for the subdomain indicated on the *x*-axis; “2” indicates medium ROB and “3” indicates a high ROB determination by the individual rater.

**Figure 3.**
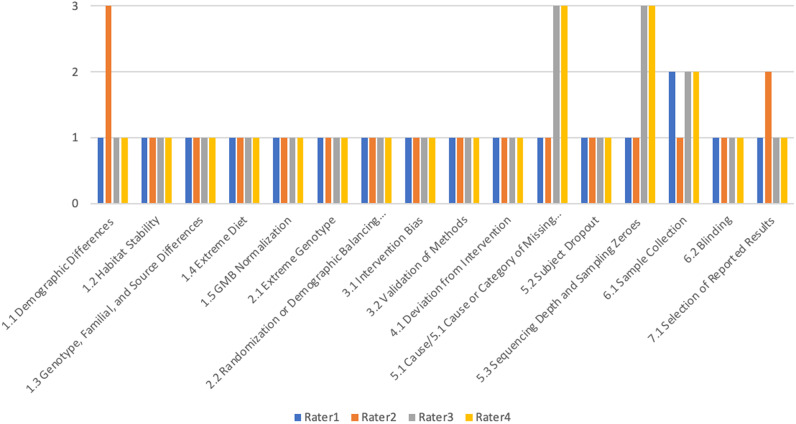
Inter-rater variability in ROB determinations by subdomain for validation test on study 3 by Saunders et al. (2020), where “1” on the *y*-axis indicates that the rater determined the study to be at low ROB for the subdomain indicated on the *x*-axis; “2” indicates medium ROB and “3” indicates a high ROB determination by the individual rater.

Inter-rater variability was assessed by assigning values of 1, 2, and 3 to low, medium, and high ROB in order to construct visual representations of rater scores in each sub-domain of bias and to compare summed ROB scores between raters for each study. Figures 1– 3 demonstrate variability within a study in each subdomain of bias assessed by this tool between raters. The figures demonstrate similar ROB judgements between at least three of four raters in the majority of subdomains across the three studies assessed.

**Figure 4.**
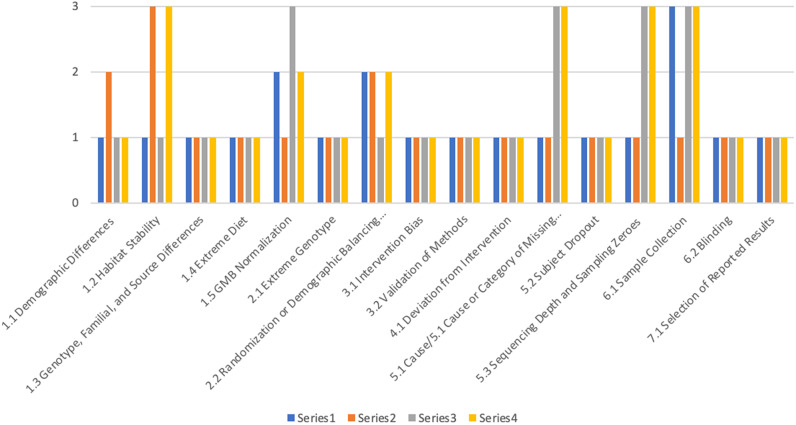
Visual representation comparing summed ROB score (as determined by assigning point values of 1, 2, and 3 to low, medium, and high ROB respectively) by rater for each of the three studies assessed in the validation test where each increasingly large concentric triangle indicates an increase of 5 points.

Figure 4 demonstrates variation in summed ROB score by rater for each of the three studies. It shows the decreasing magnitude of difference between raters’ summed ROB scores with each subsequent use of the tool from a max-score min-score difference of six points in study 1 and study 3, and of four points in study 2 out of 45 possible points. One-way ANOVA test of rater subdomain scores across all subdomains for each study returned *p*-values of 0.554, 0.568, and 0.399 for study 1, study 2, and study 3, respectively indicating no significant difference between overall ROB assessment scores between raters of the same study. First-time ROB assessors using this tool showed a relatively high degree of concordance in ROB determination at the subdomain level and in the magnitude of summed ROB score.

## Conclusion

ROB assessment is a crucial step in systematic review and meta-analysis to assess the quality of information being collected. By outlining common sources of bias that can impact GMB research following the structure of the ROBINS-I tool, this tool can serve as an adjunct to improve and standardise ROB assessment of GMB studies. A standardised ROB assessment for GMB studies will improve the accuracy of risk assessment, improve reproducibility between researchers, and promote the inclusion of high-quality information in systematic reviews and meta-analyses of the GMB.

## Data Availability

Following the journal’s policy for supporting research transparency and reproducibility, we will make all data and protocols available to readers.
